# Timing of alveolar bone surgery as a predictor of guided bone regeneration success: results of a retrospective cohort study

**DOI:** 10.25122/jml-2026-0068

**Published:** 2026-07

**Authors:** Radu Ioan Neacșu, Alexandru Metea, Horia Nițu, Bogdan Toropu, Mihai Soare, Amelia Bucur, Alina Ormenișan

**Affiliations:** 1 Faculty of Dental Medicine, George Emil Palade University of Medicine, Pharmacy, Science, and Technology, Târgu Mureș, Romania; 2 Department of Oral and Maxillofacial Surgery, Dr. Alexandru Augustin Emergency Military Clinical Hospital, Sibiu, Romania; 3 Private Dental Practice, Juillan, France; 4 Private Dental Practice, Petroșani, Romania; 5 Department of Oral and Maxillofacial Surgery, County Emergency Clinical Hospital, Sibiu, Romania; 6 Department of Mathematics and Informatics, Faculty of Sciences, Lucian Blaga University, Sibiu, Romania

**Keywords:** guided bone regeneration, collagen membrane, resorbable graft biomaterials, alveolar ridge atrophy, regional acceleratory phenomenon, predictive factor, dental extractions, complications, AB, autogenous bone, ABT, antibiotic therapy, ANOVA, analysis of variance, BSP, bone sialoprotein, BW, base width, CBCT, cone-beam computed tomography, CW, crest width, DBB, deproteinized bovine bone, GBR, guided bone regeneration, H, height, M-CSF, macrophage colony-stimulating factor, OCN, osteocalcin, OPG, osteoprotegerin, OPN, osteopontin, OR, odds ratio, RANK, receptor activator of nuclear factor κB, RAP, regional acceleratory phenomenon, SAP, systemic acceleratory phenomenon, SD, standard deviation, TNF, tumor necrosis factor

## Abstract

The physiological condition of bone prior to regenerative procedures has received little attention, with concerns more often related to technical and biomaterial issues. The primary aim of the study was to test whether pathophysiological changes in bone following tooth extractions or other alveolar bone surgery have an additional impact on bone growth in guided bone regeneration (GBR) with collagen membranes. A total of 33 consecutive patients with vertical and horizontal atrophy of the alveolar ridges, comprising 70 dental sites, treated with staged GBR using collagen membranes and a resorbable graft material, were analyzed in terms of the specific results of the procedure. The effect of defect morphology and recent bone surgery on the critical dimensions of the alveolar ridge was assessed by superimposing serial cone-beam computed tomography (CBCT) images before the augmentation procedure and after the bone healing period. Complex defects and recent bone surgeries, not older than 6 months, showed the greatest increase in crest width: 6.42 mm (*P* < 0.001) and 5.46 mm (*P* = 0.003) respectively, as well as in height: 2.80 mm (*P* = 0.007) and 1.99 mm (*P* = 0.028) respectively, while common defects and bone surgery older than 1 year led to null or negative results. Guided bone regeneration using collagen membranes was significantly influenced by the pre-existing morpho-physiological state of the bone, which could be explained by the increased osteogenic potential of the bone walls under the effect of the regional acceleratory phenomenon triggered by tooth extraction or other surgical interventions on the alveolar bone.

## Introduction

Despite its introduction into dental practice decades ago, guided bone regeneration (GBR) still raises questions about predictability, as the incidence of wound-healing complications is not negligible and can compromise the desired outcome, and the effectiveness of the procedure has been reported unevenly.

Factors involved in the occurrence of healing complications were classified as patient-, surgery-, and biomaterial-related factors [1,2]. The well-known patient-related risk factors in bone augmentation procedures, i.e., smoking, age, active periodontitis, dental condition [3], excessive graft material or trauma from temporary dentures [4], and medical conditions predisposing to infections [5], also apply to GBR. Among surgical factors, proper flap management plays an important role in preventing wound dehiscence, and operator experience is a major contributing factor [6]. As for biomaterials, several studies have shown that titanium meshes/frameworks carry a higher risk of dehiscence than membranes themselves, which in turn leads to more frequent abscess formation, with resorbable membranes performing better than non-resorbable ones in this respect [6–8].

Regarding the main objective of GBR, which is to obtain adequate bone volume for implant placement with long-term stability, specific factors were studied. Bone regeneration is hindered by medical conditions such as osteoporosis, diabetes mellitus, and low serum vitamin D levels, and conversely favored by cortical perforations, the graft’s osteopromoting properties, and membrane stabilization with pins [9–16].

The comparative regenerative potential of different bone grafts is difficult to assess due to the high heterogeneity of the membranes used in the studies [17,18]. Combining autogenous bone (AB) with a slowly resorbing synthetic or xenogeneic graft is the currently recommended approach to take advantage of both their osteogenicity and volume stability [2].

Membrane type has been shown to influence the outcomes of vertical and horizontal GBR, with shape-stable, slowly resorbable, or non-resorbable membranes yielding better results, provided healing complications are overcome [19–22]. The “sausage” technique was introduced to prevent the collapse of the collagen membrane by pushing the graft material in the crestal direction and stretching the elastic membrane over it during fixation [23].

The horizontal and vertical components of the defect are considered to have a substantial impact on the GBR outcome and the choice of the reconstruction technique. Certain treatment guidelines, depending on the type of defect, recommend the use of non-resorbable membranes for large horizontal and vertical defects and resorbable ones for horizontal defects, among other methods [24]. However, vertical ridge augmentation using collagen membranes has also been reported [21,25], raising the question of whether other factors coexist that influence the outcome of bone regeneration.

In this retrospective cohort study, we aimed to test whether the local biological state of the alveolar bone, characterized not only by morphology but also by a history of recent bone surgery, affects the predictability of GBR with a collagen membrane under a standardized protocol. Because non-resorbable bone grafts could lead to misinterpretation of radiological findings by confusing residual particles with new bone, this retrospective study included only cases in which resorbable biomaterials were used.

## Material and Methods

### Study population

The study enrolled 33 consecutive patients treated at the Department of Oral and Maxillofacial Surgery, Dr. Alexandru Augustin Sibiu Clinical Military Hospital, Romania, between March 2019 and October 2025. All surgical procedures were performed by RIN and AM. All patients were edentulous and presented with varying degrees of alveolar ridge atrophy requiring bone reconstruction before implant-supported prosthetic rehabilitation. The study included procedures that were part of the standard therapeutic protocol for alveolar bone augmentation and was approved by the hospital’s Ethics Committee.

### Inclusion criteria

- Age between 20 and 65 years, regardless of sex.
- Good general health.
- Horizontal, vertical, or combined alveolar ridge atrophy of the edentulous ridge resulting from dental or periodontal causes, which prevents appropriate dental implant placement or results in an unfavorable implant-to-crown ratio.
- Staged ridge augmentation using GBR with a collagen membrane and resorbable particulate graft material.
- Complete clinical and imaging records, including the history of dentoalveolar surgery, dental and medical status, and available CBCT examinations performed within 1 month before reconstructive surgery and 6–12 months after surgery.

### Exclusion criteria

- Medical conditions affecting bone metabolism or predisposing to infection (e.g., substance abuse, uncontrolled diabetes, immunological disorders, previous radiotherapy, ongoing chemotherapy, metabolic bone diseases, or treatment with antiresorptive agents).
- Moderate or heavy smoking (>5 cigarettes per day).
- Poor oral hygiene.
- Clinical signs of infection involving adjacent teeth (e.g., acute or subacute periapical granulomas or infrabony periodontal pockets).
- Use of any GBR membrane other than a non-crosslinked collagen membrane.
- Use of any graft material other than a resorbable biomaterial.

### Surgical procedure and postoperative care

Patients were instructed to maintain meticulous oral hygiene throughout the perioperative period and, if they were light smokers, to refrain from smoking for at least 2 weeks before and 1 month after surgery. Surgery was scheduled at least 1 month after tooth extraction at the reconstruction site to allow adequate soft tissue healing. In all cases, an antibiotic regimen consisting of 2 g amoxicillin/clavulanic acid (Amoxiplus; Antibiotice SA) administered orally 1 hour preoperatively, followed by 1 g every 12 hours for 5 days postoperatively, was prescribed.

**Figure 1:**
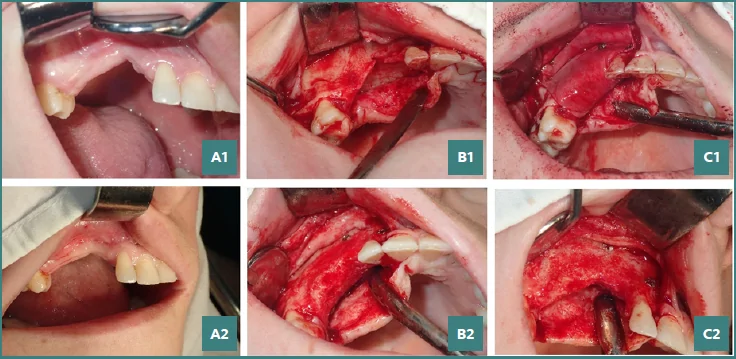
Guided bone regeneration using a collagen membrane (Osgide®) and a particulate bone graft consisting of autogenous bone and a porcine xenograft (Apatos Mix®) for the reconstruction of severe alveolar ridge atrophy 8 months after the extraction of teeth 15 and impacted 13. A1-C1, Clinical and intraoperative views of the defect and graft stabilized with an Osgide® collagen membrane fixed using Pro-fix™ pins. A2-C2, Clinical and intraoperative views of the reconstructed alveolar ridge 1 year after augmentation.

Under local anesthesia using 4% articaine with epinephrine 1:100,000 (Artidental®; Laboratorios Inibsa), a midcrestal incision was made over the edentulous ridge, extended into the gingival sulcus of the adjacent teeth, and connected to two vertical releasing incisions. A separate incision was made at the mandibular or maxillary bone harvesting site when it was not adjacent to the reconstruction site. The buccal and lingual mucoperiosteal flaps were elevated, and the deficient ridge was debrided of any residual fibrous or granulation tissue. Cortical perforations to enhance graft vascularization were created using a 1.5-mm pilot bur on each side of the alveolar ridge. However, in recent extraction sites with adequate bleeding, these perforations were minimal or omitted. Autogenous bone was harvested using a bone scraper (Safescraper Twist; Geistlich Pharma AG) and mixed in a 1:1 ratio with either nanostructured hydroxyapatite/silica gel (Nanobone®; Artoss GmbH) or a cortico-cancellous porcine xenograft (Apatos Mix®; Osteobiol®; Tecnoss), depending on graft material availability and the surgeon’s preference. An appropriately sized collagen membrane (Osgide®; Curasan) was trimmed to fit the defect and initially fixed to the lingual aspect of the ridge using 1.5 × 3-mm pins (Pro-fix™; Osteogenics Biomedical). The graft material was applied to the deficient alveolar bone and covered by the membrane, which was further fixed to the buccal side of the ridge (Figure 1). The graft material was pushed in the crestal direction under the stretched membrane before being secured with the final pins, according to the ‘sausage technique’. Periosteal releasing incisions were made in the buccal flap and, when necessary, in the lingual flap in the anterior mandibular region, with careful preservation of the mental nerve and meticulous hemostasis. Tension-free wound closure was achieved using interrupted bilayer resorbable polyglycolic acid sutures (Dacril Rapid; Biosintex).

Postoperatively, patients were prescribed nonsteroidal anti-inflammatory drugs, ice packs, and soft diet. Superficial mucosal sutures were removed on the seventh day after surgery. Clinical follow-up visits were at 24-48 hours, 1 week, 2 weeks, and 1 month postoperatively, or whenever necessary.

### Radiological measurements

**Figure 2:**
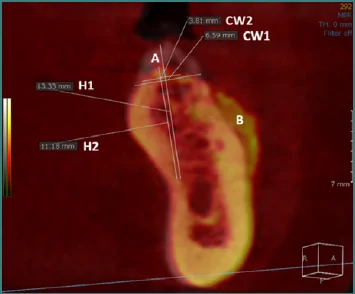
Representative measurements of ridge height (H) and crest width (CW) at 2 time points (1, baseline; 2, follow-up) after superimposition of three-dimensional reconstructed CBCT images in a patient with alveolar ridge remodeling following GBR. A = Resorbed crestal bone. B = Regenerated apical bone.

All patients underwent CBCT examination as a routine diagnostic tool before surgery and at least 6 months postoperatively. Three dimensions were measured at the vertical midline of each edentulous ridge site: height (H), basal width (BW), and crest width (CW). Three-dimensional analytical software (OnDemand3D; Cybermed) was used for image reconstruction and for measurements using the same fixed landmarks after superimposing the preoperative and postoperative datasets (Figure 2).

The fixed landmarks used for the comparative measurements in each patient were: bordering teeth of the edentulous gap (to determine the midline of each dental site in the mesiodistal direction), inferior alveolar nerve or incisive nerve, maxillary sinus floor or nasal floor (as apical landmarks in the mandible and the maxilla, respectively).

The following rules were applied: height was measured from the apical landmark to the most coronal point of the ridge, not to the base of the residual bone depression, if present. If crest height increased or decreased after reconstruction, crest width was measured 1 mm below the most coronal point of the lower crest. Basal width was measured 6 or 7 mm from the most coronal point of the lower crest, as well, depending on the available height of the crest. All primary-found values were rounded to the nearest 0.1 mm as a reasonable approximation given the image resolution and reproducibility. Because the mesiodistal size of the edentulous ridges varied, the mean value of H, CW, and BW for the dental sites composing the reconstruction area was calculated for each patient.

### Data collection and outcome variables

Hospital-level database related to the alveolar bone augmentation protocol and CBCT images constituted the main data source. The name, age, gender, and medical history, along with the biological factors considered for analysis, as well as the surgical protocol, biomaterial, medication, and postoperative course throughout the period from the GBR procedure to the reopening of the reconstruction site, were recorded in the dedicated clinical sheet.

**Figure 3:**
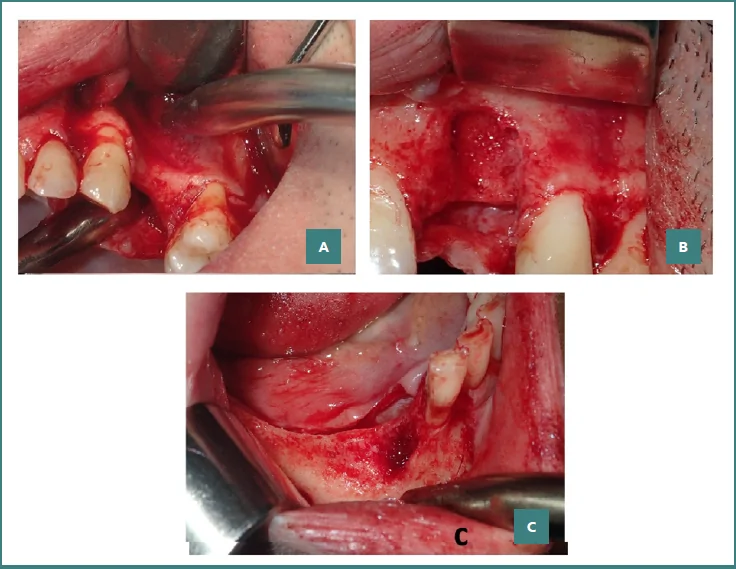
Representative examples of the defect categories used in the analysis. A, **Common defect**. B, **Complex defect**. C, **Mixed defect**.

Although the widely accepted classifications divide alveolar defects into vertical, horizontal and combined [24,26] or into defects with 1, 2 or 3 walls [2,27,28] (the latter being derived from periodontal surgery, not being specific for edentulous ridges), for simplicity and because the vast majority of the alveolar defects in everyday practice involve both vertical and horizontal dimensions, we divided atrophy of individual dental sites into two main types: *common* (approximately equivalent terms: uniform / uncontained / outside the contour) - result of bone remodeling over time, leading to a smooth-surfaced ridge and *complex* (non-uniform/contained/inside the contour), generally encountered shortly after tooth extractions, characterized by the presence of bony walls and crateriform defects. Thus, ridges were classified as common, complex, or mixed atrophy (if both common and complex atrophy were present in different dental sites that make up the edentulous ridge). Representative examples for each category are shown in Figure 3.

Two time frames for the recent surgery factor were investigated: 6 months and 1 year to compare changes in crest dimensions between recent and old ridges for each interval. Similar to classification by defect type, alveolar ridges were classified by time elapsed since last surgical intervention as *recent, old, and mixed* (whether both recent and old dental sites were present on the same ridge).

The outcome data included all types of surgical or postoperative complications, and the final dimensions (H, CW, BW) of the alveolar ridge were compared with the initial measurements obtained from the CBCT scan.

### Statistical analysis

IBM SPSS Statistics software, version 31.0.2.0(126), was used for data analysis. To avoid violating the assumption of data independence, the alveolar ridges as a whole, rather than particular dental sites (which could belong to the same patient), were analyzed, so the mean values of H, CW, and BW for the dental sites comprising each ridge were calculated. The three types of paired quantitative data (initial and final H, CW, and BW) were recorded as absolute values (mm), and differences between the initial and final conditions were presented as means, standard deviations (SD), and min-max values.

Patients were grouped by the nominal biological factors whose influence on the outcome had to be analyzed; three subgroups resulted for the recent surgery factor, for each time frame (recent, old, mixed), as well as for the defect type factor (complex, common, mixed), as previously described, and two groups for the biomaterial factor. Normality of continuous data was assessed using Shapiro-Wilk and z-tests, then Levene’s test for equality of variances of normally distributed data was interpreted.

Between-subject effects were analyzed using the independent-samples *t* test for normally distributed data with 2-group factors or one-way ANOVA followed by the Bonferroni post hoc test for multiple comparisons for 3-group factors. For nonnormally distributed data, the Mann-Whitney U test was used for 2-group factors, whereas the Kruskal-Wallis test, followed by Dunn’s post hoc test for multiple comparisons, was used for 3-group factors.

Within-subject effects were analyzed using a paired-samples *t*-test for normally distributed data, while a related-samples Wilcoxon signed-rank test was conducted for non-normally distributed data. The association between the categorical variables recent surgery and defect type was investigated using the Pearson χ^2^ test, and Lambda coefficient. A *P* value < 0.05 was considered statistically significant for between- and within-subjects effects, as well as for association analysis.

## Results

### Study population and general clinical data

**Figure 4:**
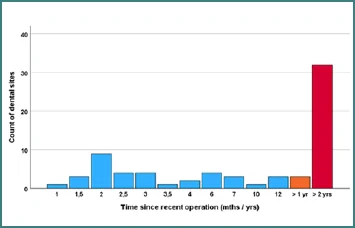
Distribution of the time elapsed since the last surgical intervention at the dental sites comprising the edentulous ridges. Blue bars represent months, and orange/red bars represent years.

A total of 33 patients with edentulous ridge atrophy of various extents, encompassing 70 dental sites, were included in the study. Table 1 summarizes demographic and clinical information, including biological factors that may serve as potential predictors of augmentation outcome. Data on recent surgical interventions (two time frames: 6 months and 1 year) and defect category were presented in the table according to the classification of the edentulous ridges described in the Methods section, while the time elapsed since the last intervention for all dental sites that make up the edentulous areas is represented in Figure 4. Most recent alveolar surgeries were tooth extractions (18 of 21), whereas the remaining procedures included implant removal, inadequate GBR, and resection of a bony concretion following bone augmentation with a nonresorbable biomaterial.

**Table 1:** Patient demographics and general clinical information on reconstruction sites

Patients’ demographics, number (%) or mean (SD), min-max		
Age (years)		44 (11), 24–63
Gender		
	male	17 (52%)
	female	16 (48%)
**Clinical information, number (%)**		
Smoking status		
	nonsmokers	31 (94%)
	light smokers	2 (6%)
Dental arch		
	maxilla	23 (70%)
	mandible	10 (30%)
Graft material		
	AB + porcine xenograft	26 (78%)
	AB + nanostructured hydroxyapatite	7 (21%)
Previous surgery – 6 months time frame*		
	recent	12 (36%)
	old	14 (42%)
	mixed	7 (21%)
Previous surgery – 1-year time frame*		
	recent	15 (45%)
	old	12 (36%)
	mixed	6 (18%)
Defect type (ridges)		
	complex	10 (30%)
	common	15 (45%)
	mixed	8 (24%)

* The same study sample was reclassified according to the two different time thresholds, so that the resulting categories do not represent separate groups of patients

### Dimensional changes in the alveolar bone after GBR procedure

Overall vertical bone gain was insignificant: 0.38 mm (SD = 2.11; *P* = 0.308), while overall horizontal bone gain, although significant, was limited for both bone levels, BW: 1.44 mm (SD = 1.44; *P* < 0.001) and CW: 2.34 mm (SD = 3.37; *P* < 0.001). However, when the effects of individual factors on the augmentation results were analyzed, both positive and negative values were obtained, as summarized in Table 2.

**Table 2:** Dimensional changes in height, base width, and crest width of the alveolar bone after the GBR procedure under the effect of the analyzed biological factors

Factor	Groups	H change (mm) mean (SD)	P*	BW change (mm) mean (SD)	P*	CW change (mm) mean (SD)	P*
previous operations 1 yr. time frame	>1 year (1)	-1.03 (1.00)	0.004	0.82 (0.86)	0.007	-0.36 (1.61)	0.458
	mixed (2)	-0.45 (1.45)	0.487	1.06 (0.84)	0.027	0.94 (1.73)	0.240
	<1 year (3)	1.84 (2.10)	0.011	2.09 (1.74)	<0.001	5.06 (2.81)	<0.001
	*P* ** (1) : (3)	<0.001		0.016		<0.001	
previous operations 6 mos. time frame	>6 months (1)	-0.66 (1.31)	0.083	1.21 (1.31)	0.004	0.33 (2.30)	0.596
	mixed (2)	-0.29 (1.39)	0.603	1.16 (0.81)	0.009	1.00 (1.58)	0.146
	<6 months (3)	1.99 (2.33)	0.028	1.88 (1.82)	0.004	5.46 (2.92)	0.003
	*P* ** (1) : (3)	0.002		0.222		<0.001	
defect type	common (1)	-1.17 (1.20)	0.002	1.15 (0.96)	<0.001	0.00 (2.03)	0.552
	mixed (2)	0.27 (1.22)	0.551	1.25 (1.40)	0.040	1.64 (1.62)	0.024
	complex (3)	2.80 (1.42)	0.007	2.03 (1.96)	0.010	6.42 (1.97)	<0.001
	*P* ** (1) : (3)	<0.001		0.327		<0.001	
graft material	porcine xenograft	0.52 (2.18)	0.234	1.54 (1.56)	<0.001	2.68 (3.52)	<0.001
	nanohydroxyapatite	-0.14 (1.91)	0.852	1.10 (0.83)	0.018	1.07 (2.56)	0.309
	*P* **	0.472		0.531		0.330	

* Within-subject comparison. **Between-subject comparison.

Regarding the height dimension, significant negative changes in H were observed in atrophies older than 1 year and in common defects; conversely, significant positive changes were observed in recently operated sites and in complex defects. Major differences were found between extreme subgroups (recent/old, common/complex) across both 1-year and 6-month time intervals and across defect types.

All BW values that changed after GBR were positive (P < 0.05), regardless of the factor analyzed, with only one significant difference found between the extreme subgroups of the 1-year time-interval factor.

Regarding the CW dimension, negative but not significant CW change values were observed in atrophies older than 1 year, whereas significant positive values were observed in recent atrophies (both 1-year and 6-month), complex defects, and porcine xenograft.

**Figure 5:**
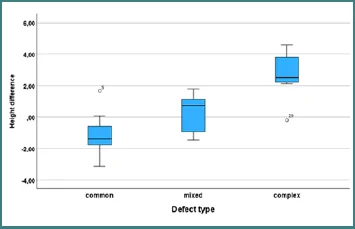
Box plot of alveolar bone height changes following GBR across the 3 defect type categories

The middle category of both previous surgery and defect type (mixed) showed values between those of the corresponding extreme subgroups for all three dimensions (Figure 5). In addition, a significant association was observed between previous surgery and defect type (Pearson χ^2^, *P* < .001), with Lambda coefficient of 0.611 for the 1-year threshold and and 0.722 for the 6-month threshold, respectively.

### Secondary findings

Healing complications were encountered in six patients (18%), of which three were major (abscesses requiring incision and ABT), and three were minor, one each from submandibular lymph node swelling, gingival fistula, and temporary labiomental hypoesthesia. Of the three abscesses, only one was related to wound dehiscence and required partial curettage of the graft material. All complications resolved after prompt surgical and medical treatment, associated with endodontic therapy of the adjacent teeth, if reactivation of periapical inflammation was suspected.

## Discussion

Differentiating the individual effects of biological factors and assessing their interactions is challenging and would require a large, homogeneous study population. One limitation of the present study is the relatively small sample size. This limitation was partially addressed by combining several statistical approaches, including comparisons of means, association analyses, and graphical representations, which allowed evaluation of the individual effects of the investigated factors. However, interactions between these factors could not be assessed because of the limited sample size and the absence of certain replicate, measurements. Another limitation of the present study is the assessment of bone size based strictly on radiological examination, which was not performed at a fixed postoperative interval due to the retrospective observational nature of the research. This approach seems more reliable in the case of bone loss than bone growth, even if resorbable materials were used, as long as the radiological examination captures just a moment of ongoing tissue remodeling and mineralization; the timing of bone growth assessment is an important issue that actually needs to be considered in histomorphometric studies as well. On the other hand, relatively homogeneous baseline characteristics, i.e. balanced distribution of gender and factor subcategories, predominance of non-smokers with only two light smokers, similar resorption time of the biomaterials used (6-12 months), along with a low incidence of healing complications, and a standardized diagnostic and therapeutic protocol support the internal consistency of the dataset and suggest that the observed outcomes are unlikely to be driven by major confounding factors.

Measuring alveolar ridge dimensions at distinct levels allowed us to differentiate critical (H, CW) from non-critical (BW) dimensional changes. Although significant bone growth was achieved in BW regardless of the variables analyzed, this was not useful for implant planning; therefore, when analyzing the efficacy of GBR with collagen membranes, we focused on the H and CW dimensions.

In the analysis of the augmentation outcome, we did not exclude the three cases with major complications, as long as two of them (one encountered at the reconstruction site and one at the bone harvesting site) healed after incision and ABT, and the other (the one associated with wound dehiscence) healed after wound debridement. It is noteworthy that only dehiscence resulted in graft failure; the other two had a positive outcome.

We proposed a simple classification of defect types based on morphology rather than vertical and horizontal components, taking into account the progressive change of alveolar ridge architecture after tooth extraction, which appeared useful for predicting GBR outcome but still needs external validation. Although defect morphology is widely recognized as an important factor in the success of GBR, to our knowledge, there is no study to date that distinguishes between the effects of complex (irregular) and common (regular) defects, or between the effects of old and recent atrophies, on the actual outcome of bone regeneration. The striking differences found in our study between these categories of the critical dimensions of the ridge (negative versus positive results) suggest that defect type and surgery timing may be potential predictive factors for the success of GBR with collagen membranes and certain resorbable graft materials. While significant positive values were obtained for both critical dimensions in complex and recent atrophies (with higher values for those not older than 6 months), not only inefficiency but even bone loss were observed in the case of common defects and old atrophies, more pronounced in those older than 1 year. This finding raises an important issue: an adverse effect of GBR in the absence of any healing complication, which has not been previously reported.

Exceptions to the general trend were found in each group, e.g. in a case of common and older than 1 year atrophy but with a large basal bone, an increase in H and CW was observed after GBR; conversely, a common and recent atrophy (< 6 months) in a multiparous woman resulted in further decrease of H and CW after GBR, and in general practice there may be various particular clinical situations, not yet thoroughly investigated.

**Figure 6:**
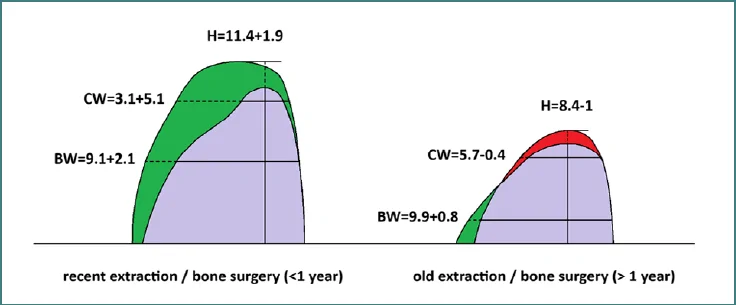
Scaled diagram of dimensional changes in the alveolar ridge after GBR, relative to the 1-year period from tooth extraction or other alveolar surgery to GBR. Green color: bone gain; red color: bone loss; W=base width; CW=crest width; H=height of the alveolar ridge, expressed as mean initial value +/- mean dimensional change

A significant association was found between defect type and recent surgery factors (both time frames), since most of the previous surgeries in the analyzed group were dental extractions. Due to particular clinical situations, the categories of defect type did not exactly overlap with those of recent surgery; the statistical results were similar for the two factors, indicating a contribution of both. A schematic representation of the effect of the recent surgery factor on the outcome of the GBR procedure is shown in Figure 6, with the defect type factor having a similar influence.

The two analyzed time thresholds were chosen based on known data on socket healing after tooth extraction, which occurs mainly in the first 6 months but may continue as remodeling even after 1 year [29]. Under such conditions, determining a time cut-off for the recent surgical factor would have been desirable, but this remains questionable given the extended period of bone remodeling.

Although not directly supported by the present study, a biologically plausible explanation for the influence of time since last surgery on GBR outcome may be found in pathophysiological changes following tissue trauma, of which the regional acceleratory phenomenon (RAP) is already well known and documented [30–32].

In bone, RAP involves physiological and biomolecular pathways that enhance healing and defense reactions after injury by increasing tissue turnover and vascularization. First, in the catabolic phase it promotes osteoclastogenesis by tumor necrosis factor (TNF)-α, macrophage colony stimulating factor (M-CSF) (secreted by osteoblasts and fibroblasts themselves), receptor activator of nuclear factor kB (RANK), osteoprotegerin (OPG), then in the anabolic phase osteoblasts display increased expression of osteopontin (OPN), bone sialoprotein (BSP), and osteocalcin (OCN) [33]. A systemic accelerator phenomenon (SAP) has also been described as playing a role in bone healing by increasing circulating OCN+ cells, which can differentiate into bone-forming osteoblasts upon homing to the site of bone repair [34,35]. Surgically induced RAP (such as after orthognathic procedures, alveolar corticotomies, or tooth extractions) is a well-documented method that accelerates orthodontic tooth movement [32,35–38]. Piezosurgery has been proposed as an adjunct procedure for GBR and site development, based on its remineralizing effect in the late postoperative phase [39]. Alveolar bone reconstruction procedures, especially early augmentation techniques such as membrane-free alveolar grafting using β-tricalcium phosphate/calcium sulfate as in situ hardening material performed 1 month after tooth extraction [40] or two-stage mandibular ridge split, with the second surgery performed 1 to 6 months after the first, according to different protocols [41–44], inherently involve RAP, although their causal relationship has not yet been investigated.

On the other hand, barrier membranes, in addition to their main role of preventing soft tissue ingrowth, have the drawback of isolating the periosteum, thus disfavoring bone regeneration by decreasing neoangiogenesis [45]. Although collagen membranes have been regarded as highly porous materials that allow the passage of nutrients into the grafted material, a recent experimental study [46] proved that they actually have a decreasing permeability to water and small solutes due to swelling and denaturation of their constituent fibers, so that after 2 weeks of immersion, they become practically impermeable, just like most other types of membranes. This suggests that guided bone regeneration occurs primarily from the underlying bone/graft material, without periosteal contribution, at least in the early stages.

The positive results of GBR when performed within 1 month to 1 year after alveolar bone surgery may be partially explained by exploiting RAP already in the anabolic phase, while increased bone vascularization compensates for the membrane’s isolation of the periosteum. In contrast, negative results in the case of old atrophic ridges are likely due to interference with the catabolic phase of RAP induced by current surgery, associated with impairment of periosteal vascularization by the artificial membrane. Therefore, the timing of GBR appears to be an additional clinical consideration when planning a GBR procedure, in addition to the properties of the biomaterials used.

In our study, the ”sausage technique” has made an important contribution to bone gain in recent atrophy and complex defects, through the stability it provides to the grafted material, necessary for bone healing; at the same time, it was not as effective in preventing apical migration of the resorbable graft material in cases of old and common atrophy. This effect has not been shown for slowly- or non-resorbable biomaterials such as deproteinized bovine bone (DBB) grafts, but clinical reports on these biomaterials have not distinguished between the two situations.

The present study provides a clinical argument for the importance of the osteogenic potential of the reconstructed site in GBR, both morphologically and physiologically, with the membrane and graft material having a supporting role.

Although the involvement of RAP in bone remodeling and healing has been documented, its contribution to guided bone regeneration has not been investigated to date. The results of our study suggest that the bone’s local physiological state influences the outcome of the augmentation procedure and warrant further exploration to help the physician integrate this into the therapeutic strategy. The findings should be interpreted with caution but provide a rationale for further prospective investigations with larger cohorts and adjusted statistical models.

## Conclusion

GBR using collagen membrane and resorbable graft biomaterials appears to be a predictable technique for complex alveolar defects (non-uniform/contained/within the contour) or recent surgical interventions on the alveolar bone, with respect to bone growth in critical dimensions (height and crest width). On the contrary, this type of augmentation technique may lead to poor results when performed for common alveolar defects (uniform/uncontained/outside the contour) or for old atrophies. RAP induced by tooth extraction or other alveolar bone surgery has the potential to be both a predictive factor and a therapeutic target in GBR procedures.

## Data Availability

The data that support the findings of this study are not publicly available due to privacy and ethical restrictions, as they contain sensitive patient information. The anonymized datasets may be available from the corresponding author upon reasonable request and with permission from the relevant institutional ethics committee.
